# Alexithymia, Pain Sensitivity, and Interoceptive Awareness in Individuals with Alcohol Use Disorder

**DOI:** 10.1192/j.eurpsy.2025.433

**Published:** 2025-08-26

**Authors:** F. Seven, M. B. Sönmez

**Affiliations:** 1 Psychiatry, Edirne Uzunköprü State Hospital; 2 Psychiatry, Trakya University School of Medicine, Edirne, Türkiye

## Abstract

**Introduction:**

Alexithymia, the limited ability to recognize and describe emotions, reflects impairments in emotional awareness and is a prevalent dysfunctional trait in individuals with addiction. Pain is an interoceptive feeling processed through interoceptive pathways and serves as a homeostatic emotion that can motivate behavior. Pain sensitivity may play a role in the development and progression of alcohol use disorder (AUD). Interoceptive awareness (IA) refers to the ability to perceive the internal state of the body. Both interoceptive accuracy (IAc) and interoceptive sensibility (IS), the objective and subjective dimensions of IA, have been shown to be implicated in individuals with AUD.

**Objectives:**

Our objective was to compare alexithymia, pain sensitivity, IAc, and IS levels between abstinent patients with AUD and healthy controls. Additionally, we aimed to investigate the potential associations between the dimensions of IA and both alexithymia and pain sensitivity.

**Methods:**

The study comprised 52 abstinent patients with AUD and 52 healthy control subjects. 92.3% (n=48) of the participants in each group were male, and 7.7% (n=4) were female. Alexithymia was assessed using the 20-item Toronto Alexithymia Scale (TAS-20). Pain sensitivity was measured with the Pain Sensitivity Questionnaire (PSQ). IAc was assessed using the heart rate tracking task, which measured participants’ awareness of their own heartbeat by comparing the number of heartbeats they perceived with an objective heart rate measurement. IS was evaluated using the Multidimensional Assessment of Interoceptive Awareness Version 2 (MAIA-2). The study included patients who had completed detoxification and been abstinent for at least three weeks while participating in or undergoing a 28-day abstinence-based inpatient treatment program.

**Results:**

Individuals with AUD scored significantly higher on self-reported measures of alexithymia (AUD group: 53.35 ± 11.83; control group: 44.63 ± 6.43; p < 0.001, F = 21.768) and significantly lower on the heart rate tracking task (IAc) (AUD group: 0.65 ± 0.15; control group: 0.84 ± 0.13; p < 0.001, F = 43.615). No significant difference was found in self-reported IS scores (AUD group: 114.06 ± 21.38; control group: 113.37 ± 13.52; p = 0.844, F = 0.039) or pain sensitivity scores (AUD group: 5.22 ± 1.67; control group: 5.18 ± 1.06; p = 0.892, F = 0.018). Alexithymia scores showed significant negative correlations with IAc scores (r = -0.256, p = 0.009) and IS scores (r = -0.361, p < 0.001). However, pain sensitivity scores did not significantly correlate with alexithymia (r = 0.083, p = 0.402), IAc (r = -0.103, p = 0.299), or IS scores (r = 0.136, p = 0.169).

**Conclusions:**

Our findings support the hypothesis that alexithymia, which is linked to the development and progression of AUD, is associated with the dimensions of IA.

**Disclosure of Interest:**

None Declared

